# An Evaluation of ChatGPT for Nutrient Content Estimation from Meal Photographs

**DOI:** 10.3390/nu17040607

**Published:** 2025-02-07

**Authors:** Cathal O’Hara, Gráinne Kent, Angela C. Flynn, Eileen R. Gibney, Claire M. Timon

**Affiliations:** 1 School of Population Health, Royal College of Surgeons in Ireland (RCSI), D02 YN77 Dublin, Ireland; 2 UCD Institute of Food and Health, University College Dublin, D04 V1W8 Dublin, Ireland; 3 School of Agriculture and Food Science, University College Dublin, D04 V1W8 Dublin, Ireland

**Keywords:** dietary intake assessment, image-based dietary intake assessment, food composition, generative artificial intelligence, computer vision, large language models

## Abstract

**Background/Objectives**: Advances in artificial intelligence now allow combined use of large language and vision models; however, there has been limited evaluation of their potential in dietary assessment. This study aimed to evaluate the accuracy of ChatGPT-4 in estimating nutritional content of commonly consumed meals using meal photographs derived from national dietary survey data. **Methods**: Meal photographs (*n* = 114) were uploaded to ChatGPT and it was asked to identify the foods in each meal, estimate their weight, and estimate the nutrient content of the meals for 16 nutrients for comparison with the known values using precision, paired *t*-tests, Wilcoxon signed rank test, percentage difference, and Spearman correlation (r_s_). Seven dietitians also estimated energy, protein, and carbohydrate content of thirty-eight meal photographs for comparison with ChatGPT using intraclass correlation (ICC). **Results**: Comparing ChatGPT and actual meals, ChatGPT showed good precision (93.0%) for correctly identifying the foods in the photographs. There was good agreement for meal weight (*p* = 0.221) for small meals, but poor agreement for medium (*p* < 0.001) and large (*p* < 0.001) meals. There was poor agreement for 10 of the 16 nutrients (*p* < 0.05). Percentage difference from actual values was >10% for 13 nutrients, with ChatGPT underestimating 11 nutrients. Correlations were adequate or good for all nutrients with r_s_ ranging from 0.29 to 0.83. When comparing ChatGPT and dietitians, the ICC ranged from 0.31 to 0.67 across nutrients. **Conclusions**: ChatGPT performed well for identifying foods, estimating weights of small portion sizes, and ranking meals according to nutrient content, but performed poorly for estimating weights of medium and large portion sizes and providing accurate estimates of nutrient content.

## 1. Introduction

The assessment of dietary intake is an essential task in identifying and managing food and nutrition-related causes of poor health at both the individual and the population level [1]. The most commonly used methods of dietary intake assessment rely on people reporting the foods, food groups, or meals that they consume and their respective portion sizes. This information can then be used together with food composition databases to estimate the quantity of nutrients or other food components consumed [2].

The use of digital technology in dietary intake assessment has increased in recent decades with digital versions of food diaries, 24-h recalls, and food frequency questionnaires now widely available [3]. These digital methods are typically web- or app-based and rely on users typing the foods and drinks that they consume and selecting suitable matches from a list [3,4]. To reduce the user burden and potentially improve accuracy, many systems now also include image recognition software, allowing users to upload photographs of their dietary intakes rather than searching for individual foods; artificial intelligence can then be used to automatically recognize the foods in the meal photographs and estimate portion sizes and nutritional content [5,6,7].

In addition to the availability of digital methods of dietary intake assessment, the general public are also increasingly using the internet to source information about nutrition [8,9,10]. However, the way in which people interact with digital technology and online information is changing rapidly, especially since the public launch of OpenAI’s large language model (LLM), ChatGPT, in November 2022 [11]. Many other models, such as Google’s Gemini and Microsoft’s Copilot, have also been launched [12]. These LLMs act as powerful chatbots, providing human-like text responses to typed queries from users [11,12]. Following recent advances, many of these models are now multimodal, i.e., in addition to text, they can also work with a variety of different types of media, including images, video, and audio [12].

While these new tools present an opportunity for dietary intake assessment, the limited research evaluating their use for this purpose has shown variable accuracy. One study used text-based queries to ask four different LLMs to classify foods as either low or high in potassium and phosphorous. Between 66% (ChatGPT3.5) and 81% (ChatGPT4 and Bing Chat) of foods (*n* = 149) were correctly classified for potassium and between 77% (ChatGPT4) and 100% (Bard AI) of foods (*n* = 81) were correctly classified for phosphorous [13]. Another study, also using text-based queries, assessed the ability of ChatGPT3.5 to estimate the energy, macronutrient, and water content of 236 different food items. The percentage of foods that had an error of <10% ranged from 29.9% for fat to 66.4% for energy [14]. A study by Lo et al. [15] is the only study identified to date that used the image recognition function of ChatGPT for nutrition purposes. They reported that ChatGPT accurately identified the foods in 16 photographs of Ghanaian and Kenyan meals and accurately estimated portion sizes. However, micronutrient content was not considered, and a limited number of evaluation metrics were reported for the estimates of energy and macronutrients, making it difficult to evaluate the overall accuracy for nutrition estimations [15].

The existing research on LLMs for use in dietary intake assessment is limited, with existing studies focusing on using text-based information only, examining a small range of nutrients, or focusing on meals of specific regional origin. Further research is required to broaden the research base and understand the capabilities of LLMs in dietary intake assessment. The aim of the current study was to evaluate ChatGPT for use as a tool to identify foods in meal photographs representative of those consumed in a national dietary survey, estimate the portion sizes of those meals, and estimate their nutritional content.

## 2. Methods

### 2.1. Ethics Statement

The current study was conducted according to the guidelines of the Declaration of Helsinki and approved by the Human Research Ethics Committee of the Royal College of Surgeons in Ireland (reference number: REC202405012, date of approval: 9 July 2024).

### 2.2. Meal Images

The meal images used in this study were photographs of 38 meals that are representative of those consumed during the National Adult Nutrition Survey (NANS) (2008–2010) in Ireland [16]. These meals have been identified and photographs taken during previous research, as described elsewhere [17,18]. In brief, there are 3 photographs of each meal (a total of 114 photographs) depicting 3 different portion sizes, representative of the portion sizes consumed by participants from NANS. The photographs represent four meal types: breakfast, lunch, dinner, and snacks. Photographs were taken using a standardized process without any background objects in the images except for knives, forks, or spoons, depending on the meal being photographed. When the meals were prepared, each food was weighed and its nutrient composition determined using McCance and Widdowson’s Composition of Foods via Nutritics version 6.04 (Dublin, Ireland). The meals varied in complexity; the number of foods in the meals ranged from one (e.g., a piece of cake) to nine (e.g., a salad meal with an accompanying drink). For some meals, all foods were clearly visible in the image, whereas for others, some foods were hidden by others in the image, e.g., in soups, sandwiches, or salads. Examples of the meal photographs are provided in Figure S1 of the Supplementary Materials.

### 2.3. Nutrition Estimation by ChatGPT

ChatGPT-4 was used for the study, and all prompts were provided by the researcher (C.O’H.) while logged in from the same account. A new chat was opened, and the following prompt was given to ChatGPT:

“I have some photos for which I want you to provide me with an estimate of the nutritional content. Specifically, I want you to give me an estimate of energy, protein, total carbohydrate, total fat, dietary fibre, total sugar, saturated fat, polyunsaturated fat, monounsaturated fat, calcium, iron, vitamin D, sodium, potassium, folate, folic acid, and vitamin C in the total meal. Please also provide a list of the foods in the photo with an estimate of the physical weight of each food in grams. Please provide your best point estimate and do not provide a range. When estimating the weight, please provide a weight estimate for each individual ingredient you identify in the meal rather than the combined weights of multiple ingredients.”

After the prompt, a single meal photograph was uploaded. ChatGPT’s response to that photograph was recorded; this included the individual foods that ChatGPT identified in the meal, their weights, the nutritional composition of the meal for the 16 nutrients, and any additional comments. This process was repeated for the other photographs. Photographs of meals with a medium portion size were completed first in a single chat. A new chat was then opened for photographs with a small portion size, and finally, a third chat was opened for photographs with a large portion size. The same prompt and process as described above was used for all portion sizes. ChatGPT was asked to provide an estimate for each photograph once only. This process was devised based on the multidisciplinary experience of the research team to ensure that the appropriate data were available for the planned analysis. The nutrients were chosen because they are of importance to public health in Ireland [19,20].

### 2.4. Recruitment of Dietitians

Previous studies comparing different image recognition software with dietitians used a sample size ranging from two to eight [21,22,23]. Given the exploratory nature of this research, the limited research in the area, and based on previous studies, the aim was to recruit between five and ten dietitians. The eligibility criteria included CORU-registered or HCPC-registered (these are the statutory regulators of health and social care professionals in Ireland and the UK, respectively) dietitians who were practicing in Ireland or the United Kingdom at the time of participation, and who, as part of their work, were involved in estimating the nutritional content of foods, meals, or dietary intakes. Recruitment was carried out via email and the LinkedIn social media platform among professional contacts of the researchers and distributed by Irish Nutrition and Dietetic Institute (INDI) via email to their members.

### 2.5. Nutrition Estimation by Dietitians

Data were collected via an online anonymous questionnaire using REDCap electronic data capture tools version 13.5.4 hosted at the Royal College of Surgeons in Ireland [24,25]. A link to the survey was included in all recruitment material. Those who clicked on the link were presented with the study information and electronic consent form. Informed consent was obtained from all participants.

The survey consisted of three sections. The first section checked eligibility of potential participants. The second section collected data on participants’ professional role, experience with dietary intake assessment, and demographic details. In the third section, participants were presented with the meal photographs one after the other and for each one they were asked to estimate the nutrient content. To reduce respondent burden, dietitians were only asked to estimate the nutritional content of the medium portion sizes of the meals, i.e., a total of 38 photographs. Additionally, the nutrients they were asked to estimate were limited to energy, protein, and carbohydrate; these nutrients were chosen based on the authors’ clinical experience of the nutrients most commonly assessed by dietitians in practice. Dietitians were asked to provide their best quick estimate without consulting external sources. Following the estimation of the nutritional content, there was a free-text question asking dietitians about what other information they would require to provide more accurate estimates.

### 2.6. Statistical Analysis

#### 2.6.1. Analysis of Image Recognition

Each meal is comprised of a number of individual foods. Foods in the meal photographs correctly identified by ChatGPT were labelled true positives (TP), those that were in the photographs but not identified by ChatGPT were labelled false negatives (FN), and foods ChatGPT incorrectly reported to be present in the actual meal when they were not were labelled false positives (FP). In the case of true positives, approximate matches were included in this value to account for the lower level of detail provided by ChatGPT when describing foods; for example, “milk in tea” described by ChatGPT was considered a match with “milk, whole, pasteurised, average” as described in the McCance and Widdowson’s Composition of Foods. This approach is consistent with previous studies evaluating digital methods of dietary intake assessment [26]. Precision is the ability of the model to identify foods present without suggesting foods that were not present; this was calculated as $\frac{TP}{TP+FP}$. Recall is the ability of the model to identify foods present without missing any foods; this was calculated as $\frac{TP}{TP+FN}$. The F1 score combines precision and recall using the harmonic mean, and was calculated as $\frac{2}{Recall^{-1}+Precision^{-1}}$.

#### 2.6.2. Analysis of Portion Sizes

The gram weights for each of the individual foods in each photograph estimated by ChatGPT was summed to give a meal weight for each photograph. The mean and standard deviation of estimated meal weights were calculated separately for meals with small portion size, those with a medium portion size, and those with a large portion size. The weights from ChatGPT were compared with the actual weights separately for each portion size (small, medium, and large) using a paired-samples *t*-test (data were deemed to be normally distributed following Shapiro–Wilk tests). *p*-values of <0.05 indicated poor agreement while those with *p* ≥ 0.05 indicated good agreement [27].

#### 2.6.3. Comparison of ChatGPT Estimates with Actual Nutrient Content

Summary data of the nutrient content of the meals estimated by ChatGPT, the actual content, and comparisons between the two are presented as absolute values for all nutrients and energy-adjusted values for the energy-containing nutrients, i.e., the percentage of the total energy content of meals from those nutrients (% energy). The distributions of the nutrient content data did not follow a normal distribution as per the Shapiro–Wilk test, so data were summarized using median and interquartile range, and Wilcoxon signed rank test and Spearman correlation were chosen as non-parametric methods. The estimates of nutrient content from ChatGPT were compared with the actual content using percentage difference, Wilcoxon signed rank test, Spearman correlation, and cross-classification of quartiles. A percentage difference of greater than ±10% indicated poor agreement, *p*-values of <0.05 indicated poor agreement, and those ≥0.05 indicated good agreement; correlation coefficients of <0.20 indicated poor agreement, coefficients ≥ 0.20 and <0.50 were deemed acceptable, and those ≥0.50 indicated good agreement; for cross-classification of quartiles, exact agreement of ≥50% was considered good, and extreme disagreement of <10% was also considered good [27]. Wilcoxon effect size (r) ≥ 0.1 and <0.3 was considered small, ≥0.3 and <0.5 was considered moderate, and ≥0.5 was considered large [28]. Adjustments were not made for multiple comparisons given the exploratory nature of the study and to balance the risks of type 1 and type 2 errors in the context of the study exploring a potential new method of dietary intake assessment.

#### 2.6.4. Comparison of ChatGPT Estimations with Dietitians

For estimation of energy, carbohydrate, and protein content of meals, intraclass correlation (ICC) (two-way random effects, single rater, absolute agreement model) was conducted to assess the agreement among dietitians and ChatGPT, i.e., the agreement among eight raters including seven dietitians and ChatGPT as an additional eighth rater. ICC coefficients of <0.5 were considered poor, ≥0.5 and <0.75 were considered moderate, ≥0.75 and <0.9 were considered good, and ≥0.9 were considered excellent [29].

## 3. Results

### 3.1. Image Recognition

There was a total of 547 foods present (including duplicates) across the 114 meal photographs. There were 463 true positives, 35 false positives, and 84 false negatives. This gave rise to a precision of 93.0%, a recall of 84.6%, and an F1 score of 88.6%.

### 3.2. Portion Sizes

**Figure 1:**
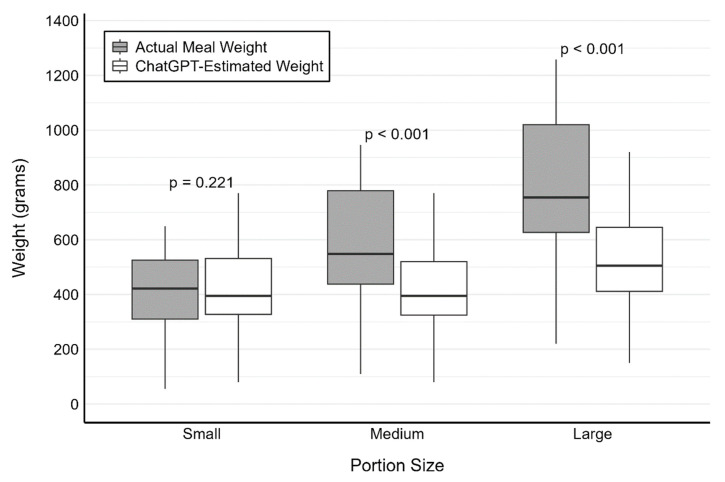
The distribution of meal weights for 3 different portion sizes of 38 meal photographs comparing the actual weights of the meals with the weights estimated by ChatGPT. *p*-values are based on a paired *t*-test between actual values and ChatGPT values.

The mean ± SD of the actual meal weights and the ChatGPT-estimated weights, respectively, were 408.2 g ± 155.0 g and 430.5 g ± 166.7 g (*p* = 0.221) for small portion size meals, 580.5 g ± 213.8 g and 425.8 g ± 165.9 g (*p* < 0.001) for medium portion size meals, and 798.1 g ± 255.3 g and 529.5 g ± 167.9 g (*p* < 0.001) for large portion size meals. The distributions of meal weights are presented in Figure 1. Across all meal photographs, the absolute percentage difference in meal weight ranged from 0.2% to 100%, with a mean ± SD absolute percentage difference of 27.8% ± 18.5%. ChatGPT underestimated the weight for 87/114 (76.3%) of the meal photographs.

### 3.3. Comparison Between ChatGPT and Actual Nutrient Content

Summary values for the actual nutritional content for 16 nutrients and ChatGPT estimates for those nutrients in 114 meal photographs are shown in Table 1. The percentage difference between ChatGPT and the actual nutrient content was less than ±10% for energy in kilocalories (0.1%), protein in grams (−2.7%), carbohydrate in grams (−6.5%), and polyunsaturated fat in grams (−9.1%), with negative values indicating underestimates by ChatGPT. The remaining nutrients had a percentage difference of greater than ±10%, with ChatGPT providing underestimates for 11 of the 16 nutrients. The mean absolute percentage difference was 26.9%. There was a statistically significant difference (*p* < 0.05) between ChatGPT-estimated and actual nutrient content for 10 of the 16 nutrients. The effect size was small for nine nutrients, moderate for three, and large for four. The comparisons using energy-adjusted units were not markedly different from those that were not energy-adjusted (Table 1).

Correlation coefficients between actual nutrient content and the ChatGPT estimates for the 16 nutrients ranged from 0.29 to 0.83 with a mean coefficient of 0.62. Vitamin D was not included in the cross-classification of quartiles due to the number of meals containing no vitamin D. For the remaining fifteen nutrients, the exact agreement ranged from 33.3% to 57.9% with a mean value of 46.8% and eight nutrients having a value of ≥50%. No nutrients had extreme disagreement for >10%, with values ranging from 0.0% to 8.8% and a mean value of 2.1%. The comparisons using energy-adjusted units were not different from those that were not energy-adjusted (Table 2).

### 3.4. Description of Dietitians

Seven dietitians took part in the study. Five were based in Ireland (71.4%) and two were based in the UK (28.6%). They had a mean ± SD dietetics experience of 6.9 years ± 4.7 years.

Six dietitians (85.7%) reported that they estimated the nutritional content of foods or meals on either a daily (*n* = 3, 42.9%) or weekly (*n* = 3, 42.9%) basis in general, and specifically for the purposes of estimating energy or protein content, *nn*; one (14.3%) reported doing this less than weekly. Two dietitians (28.6%) reported estimating the carbohydrate content of foods or meals on a weekly basis, two (28.6%) reported this less than weekly, while three (42.9%) reported that they never undertook this task.

**Figure 2:**
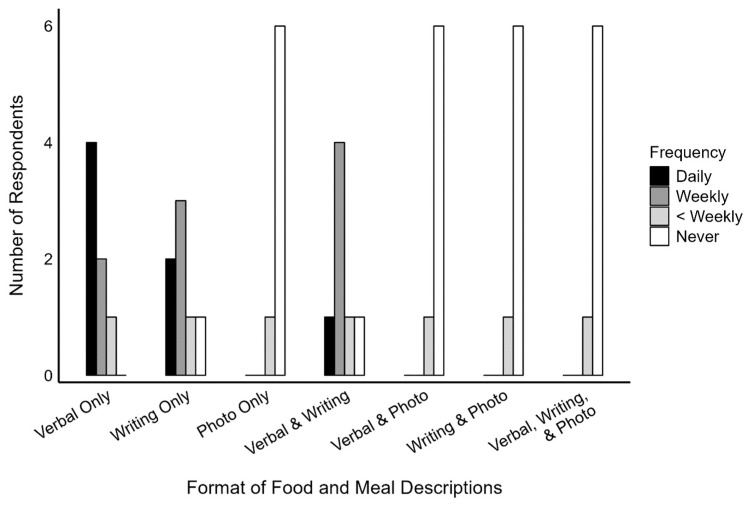
The format of food and meal descriptions used by the dietitians (*n* = 7) when estimating the nutritional content of those foods and meals in their daily practice.

Dietitians reported that when estimating the nutritional content of foods or meals in their daily practice, they most commonly do this based on verbal and/or written descriptions of those foods or meals. Only one dietitian (14.3%) reported using photographs for this purpose (either alone or in combination with verbal and/or written descriptions), and they did so less than weekly (Figure 2).

### 3.5. Agreement Among ChatGPT and Dietitians’ Estimates

Four dietitians (57.1%) provided an estimate for all three nutrients requested for all thirty-eight meal photographs. Three dietitians (42.9%) did not provide any responses for one of the meal photographs, i.e., they provided responses for thirty-seven of the meals. It was a different meal that was missed by each of those dietitians. Missing values were excluded from the analysis. There were estimates from ChatGPT for all meals.

The ICC for energy content was 0.56 (*p* < 0.001, 95% CI: 0.38 to 0.72), indicating poor to moderate agreement. The ICC for protein content was 0.67 (*p* < 0.001, 95% CI: 0.53 to 0.80), indicating moderate to good agreement. The ICC for carbohydrate content was 0.31 (*p* < 0.001, 95% CI: 0.16 to 0.49), indicating poor agreement. There were no significant changes to the ICC values when the analysis was repeated without ChatGPT.

### 3.6. Additional Comments Provided by ChatGPT and Dietitians

While the prompt used in the current study did not specifically request any additional information about the nutrition estimations, ChatGPT did make comments providing information on the limitations and assumptions of those estimations. For example, it referred to the potential for its estimations to differ from the actual content depending on whether certain foods in the meals were fortified, making statements such as “cornflakes can often be fortified with vitamins and minerals, which would significantly affect their nutritional profile, especially for iron and B vitamins”. Other comments highlighted uncertainties about portion size: “the caloric estimate assumes the butter is spread thinly; if more butter is used, the fat and calorie content could be higher”. For another meal, it suggested weighing the foods: “for the most accurate information, it would be best to use a digital scale to weigh each food item and a nutritional database for exact brand and preparation methods”. There were also comments about uncertainties related to cooking methods, unseen ingredients, and food brands in the images: “for more accurate nutritional details, the exact type of each food, brand, recipe, and any added ingredients should be considered”.

Dietitians were specifically asked to suggest what additional information could improve their estimations. The responses provided in this free-text question were similar to the comments made by ChatGPT. The additional information suggested in the responses included, for example, “type of fillings, cooking methods, types of spread/milk”, “written descriptions of what exactly was in foods such as sandwiches and salads”, “cooking methods, ingredients, weights”, and it was also stated that “written or verbal information would help”.

## 4. Discussion

The current study used both the LLM and image recognition functions of ChatGPT-4 to evaluate its ability to estimate the nutritional composition of 114 meal photographs derived from national dietary intake survey data. ChatGPT correctly identified the majority of the foods in the photographs. However, portion size estimations of the meals were only accurate for smaller meals, with ChatGPT underestimating the weight of medium and large meals. ChatGPT also underestimated the content for most nutrients included in this study. However, correlation and cross-classification of quartiles indicated good ability of ChatGPT to rank meals according to nutritional content. It performed comparably to dietitians, with the intra-class correlation ranging from poor to good depending on the nutrient, with the best agreement for protein, followed by energy, and then carbohydrate.

Only one previous study used ChatGPT to estimate the nutritional content of meal photographs. That study used photographs from the free-living environment, but they were not representative of meals consumed in any national dietary survey. Those researchers reported similar results to the current study for precision, recall, and F1 score (84.1%, 88.1%, and 86.1%, respectively) for the ability of ChatGPT to identify foods from photographs [15]. While the aim of the current study was to provide a baseline evaluation of ChatGPT in dietary assessment rather than test different prompting or fine-tuning strategies, Lo et al. [15] reported an increase of at least six percentage points for precision, recall, and F1 score by using a prompt that provided contextual information about the type of cuisine in the photographs. Future work should consider a variety of prompting strategies to identify the optimal approach. Another study used the ChatGPT LLM with a separate image recognition model to create a chatbot for people with type 2 diabetes [30]. They assessed the ability of image recognition for 11 carbohydrate containing foods, finding an average F1 score of 82.5%. Other studies that have tested image recognition models specifically designed for food recognition have achieved values of >90% for precision, recall, and F1 score [31,32,33]. These models, however, have been tested on food image datasets with thousands or tens of thousands of photographs, making it difficult to compare with the results of the current study in which 114 photographs were used. Despite this, the relatively high values for the performance metrics in the current study highlight the potential for ChatGPT in food image recognition; however, further research is required that utilizes different fine-tuning and prompting strategies, as well as testing on larger food image datasets.

Following on from identifying foods in the meal images, portion size was estimated by ChatGPT. While the current study examined portion size at the meal level, Lo et al. [15] examined the ability of ChatGPT to estimate the portion sizes of individual foods within meals. In contrast to the results of the current study, where there was good agreement for meals with a small portion size but not for medium or large meals, Lo et al. [15] reported minimal variation between the ChatGPT estimates and the true values for all foods. In a review of image-based food recognition, the percentage error in portion size estimation by models specifically designed for food image recognition was reported to range from 1.1% to 85% [7], which is comparable to the results of the current study. The portion sizes in the current study were based on the portion sizes actually consumed by participants in the National Adult Nutrition Survey in Ireland [16] as opposed to the recommended portion sizes given in food-based dietary guidelines (FBDGs). Given that the ChatGPT model is trained on large volumes of data available in the public domain [11], it is possible that its portion size estimations were influenced by portion size recommendations from FBDGs. Those portion recommendations tend to be smaller than the portions present in the meal photographs used in the current study; for example, recommended portions in various countries for ready-to-eat cereals range from 18 to 60 g, whereas the ready-to-eat cereals in the photographs in the current study ranged from 30 to 70 g [34]. This was similar for other food groups also, such as potatoes (50–250 g in FBDGs versus 180–360 g in current study) and meat (28–200 g in FBDGs versus 42–265 g in current study) [34]. However, further work is required to determine to what extent this influences ChatGPT’s portion size estimations. Another source of potential error in ChatGPT’s portion size estimation is its ability to calibrate itself based on the specific contents of each individual photograph. Generally, food image recognition models require the photograph to include objects of known dimensions such as a fiducial marker, a coin, or the user’s thumb etc., for calibration, which enhances the accuracy of portion size estimation [5,6,7]. When evaluating ChatGPT for portion size estimation, Lo et al. [15] specifically prompted ChatGPT to use background objects in the photograph as references for approximating portion sizes but did not evaluate whether this prompt improved accuracy. The photographs in the current study were taken using a standardized process, excluding any background objects with the exception of cutlery. While it is possible that ChatGPT incorporated the presence of cutlery into its portion size estimations, this was not specifically requested in the prompt used in the current study. Future work should consider the use of fiducial markers or different prompting strategies to determine their impact on the accuracy of portion size estimation by ChatGPT.

It is likely that the underestimation of portion size in the current study will have influenced the estimations of nutrient content, which were also, on average, underestimated by ChatGPT. The current study found that adjusting the nutrient content data relative to energy content of the meals did not impact on the overall comparison, indicating that both energy and other nutrients were underestimated to a similar degree. This is similar to the findings from Lo et al. [15], who reported that meals with less accurate ChatGPT estimations of portion size also had less accurate estimates of energy, carbohydrates, protein, and fat. However, they did not report whether there were trends towards either over or underestimation. Lu et al. [23] also reported similar findings to the current study in their assessment of a food-specific image recognition model, with correlations between estimations from the model and actual values for energy and macronutrients ranging from 0.50 to 0.89. The food image recognition model used in another study achieved correlation coefficients of >0.70 for protein, fiber, vitamin C, calcium, and iron [35], representing better agreement for the micronutrients than that seen in the current study. Using a model designed to estimate the carbohydrate content of meals from photographs, Vasiloglou et al. [20] reported a correlation of 0.76 between image recognition estimates and actual values, which was higher than the value of 0.59 reported in the current study. In a recent systematic review, Shonkoff et al. [36] reported that the average relative error for estimation of calories across 20 studies ranged from 0.1% to 38.3%, which is in keeping with the equivalent value of 27.3% observed in the current study. While not reported elsewhere in the literature, results from the current study showed generally better agreement for energy and macronutrients compared with micronutrients. For example, six of the eight highest effect sizes for the difference between ChatGPT and actual were micronutrients and five of the eight highest percentages differences were also micronutrients. It is possible that this relates to the more variable micronutrient content of foods due to the potential for varying degrees of fortification.

One of the major differences between the models that are specifically designed for food image recognition purposes and ChatGPT is that those other models can directly access food composition databases [5,6,7], potentially providing greater accuracy for nutrient estimations. ChatGPT, however, does not directly access these databases when responding to queries; instead, its responses are based on the probability of various words occurring together in sentences [11]. This has implications for its role in dietary intake assessment. One potential way of overcoming this limitation is the use of retrieval augmented generation (RAG) to incorporate nutrition databases with LLMs. RAG combines retrieval and generation whereby the model can retrieve data from specific databases and generate answers based on those retrievals [37]. While there is limited evidence for its use in dietary assessment, it has been used to improve the ability of an LLM to summarize nutrition information from electronic health records [38]. It is possible, therefore, that food composition databases—and indeed other sources of nutrition knowledge—could be integrated into LLMs designed for dietary assessment, allowing them to provide more specific and factual information.

The current study also examined the inter-class correlation among ChatGPT and 7 dietitians for estimating energy, protein, and carbohydrate from 38 meal photographs. The correlation ranged from poor to good depending on the nutrient, with the best agreement for protein, followed by energy, and then carbohydrate. The correlations did not change significantly when the analysis was repeated without ChatGPT. Only a limited number of studies have compared the estimation of the nutritional content of meal photographs by dietitians with that of artificial intelligence. In one study, photographs of meals were sent to three dietitians via an app who then estimated their energy and macronutrient content using a nutritional composition database. These dietitians had been specifically trained and had experience in conducting dietary intake assessments using meal photographs. ICC values were higher in that study, ranging from 0.48 to 0.93 [39] compared to the current study (0.31 to 0.67). In another study, two photographs of each meal were taken at different angles with a credit card-sized reference card next to the meals. Dietitians estimated the carbohydrate content of these meals based on food composition data. The dietitians involved in this study worked specifically with people with diabetes and had experience of carbohydrate counting. An ICC of 0.86 was reported for the dietitians [22]. There are several reasons for the differences observed between the current study and those previous studies. In the current study, no specific training was provided to the dietitians to estimate nutritional content from meal photographs, they were not provided with specific nutrition composition databases and were specifically instructed not to use one, and no fiducial markers were included in the photographs like those previous studies. While all of the dietitians in the current study regularly conducted dietary assessments, only one of the dietitians used photographs for this purpose. This approach was deemed appropriate for the current study because the comparison was made with a general-purpose language and image recognition model rather than one that was specifically designed for use in nutrition and dietetics. Futures studies should consider fine-tuning ChatGPT for nutrition purposes, including access to food composition data and comparing with dietitians with training and experience with dietary assessment specifically using meal photographs and who have access to the composition data.

ChatGPT was used “as-is” in the current study, without any fine tuning applied to enhance its performance for use specifically in dietary intake assessment. Given the increasing reliance on online sources of nutrition information [8,9,10], it was considered important to evaluate ChatGPT in the format in which it is currently available to the general public. While the presence of inaccurate or misleading nutrition information online is not a new phenomenon, LLM’s have the potential to make this information more widely available, representing a possible risk to public health [40,41]. LLMs typically provide responses using a confident and authoritative tone [40,41]; however, they can also, as observed in the current study, provide warnings about the limitations and assumptions of their responses. However, even with the inclusion of such warnings, it is possible that people will consider the portion size and nutrient content estimations to be accurate, despite systematic errors. Further work is needed to develop ChatGPT and other LLMs to be fit for purpose if used for image-based dietary intake assessment.

In addition to the limitations of this study that have been noted throughout the discussion, there are other limitations that have not yet been highlighted. The examination of the reproducibility of the responses provided by ChatGPT and the impact of any fine-tuning or different prompting strategies was beyond the scope of the current study. While the results of the current study provide a baseline evaluation of ChatGPT’s performance, this performance may change over time due to model updates, different prompting strategies, or influences of users’ previous prompts from the same account [12]. The authors acknowledge that these are important factors and require detailed investigation in future studies. While the aim of the current study was to provide a detailed account of one publicly available LLM, ChatGPT, future research is also needed to replicate this with other LLMs and to conduct direct comparisons among them. A relatively small number of meal photographs were used to evaluate ChatGPT’s performance. However, a strength of the current study is the high quality and detailed nutritional information associated with those photographs, allowing for the assessment of a range of different nutrients. This field of research is limited by a lack of large meal image datasets with associated nutritional information for a wide range of nutrients and further work is required to develop such datasets [6].

## 5. Conclusions

ChatGPT-4 showed good performance for identifying foods from photographs of meals, estimating the weights of meals with a small portion size, and ranking meals according to their nutritional content. However, it underestimated the weight of medium and large meals and consequently underestimated nutrient content for most nutrients. Given that ChatGPT is a general purpose LLM, as opposed to one specifically designed for use in nutrition and dietetics, these results demonstrate good potential for LLMs to be used in dietary assessment. Further research is needed to train these models for use in nutrition and dietetics and to link them with relevant nutrition databases, expanding their use beyond just assessing the nutritional content of individual meals and also examining their feasibility and accuracy for use in more comprehensive methods of dietary intake assessment such as food diaries, 24-h recalls, and diet histories. Further work will also be required to determine how such tools could fit within existing nutrition and dietetic workflows and to examine the acceptability of these tools to nutritionists, dietitians, patients, and the general public.

## Figures and Tables

**Table 1:** Comparison of median nutrient content of 114 meal photographs with ChatGPT-estimated nutrient content.

Nutrient	Nutrient Content, Median (IQR)		Difference, %	*p* Value	Effect Size, r	Effect Size, Magnitude ^1^
	Actual	ChatGPT				
Energy (kcal)	524.5 (361.5–740.5)	525 (350–700)	0.1	0.219	0.115	small
Protein (g)	25.7 (11.8–40)	25 (12.8–35)	−2.7	0.001	0.304	moderate
Protein (% energy)	18.6 (11.6–27)	18.6 (11.4–23.9)	−0.3	0.002	0.294	small
Carbohydrate (g)	62 (43.2–84.8)	58 (40–75)	−6.5	0.007	0.251	small
Carbohydrate (% energy)	46.1 (40.8–55.3)	41.8 (34.1–48.9)	−9.3	0.014	0.232	small
Fat (g)	16.6 (8–27.9)	19 (10–25)	14.8	0.446	0.069	small
Fat (% energy)	27.4 (16.2–37)	33.2 (24.4–41.4)	21.3	0.016	0.226	small
Dietary Fibre (g)	6.2 (4.2–9.2)	5 (2–7.9)	−20.0	0.000	0.504	large
Sugar (g)	11.8 (6.2–20.1)	8 (5–24)	−32.2	0.003	0.281	small
Sugar (% energy)	8.9 (4.5–18)	5.7 (4.1–15.8)	−36.3	0.002	0.285	small
Saturated Fat (g)	5.3 (2.5–9.7)	7 (3–10)	30.8	0.335	0.090	small
Saturated Fat (% energy)	8.8 (6–13.8)	11.2 (6.1–15.4)	27.7	0.259	0.106	small
Polyunsaturated Fat (g)	2.2 (1.2–4)	2 (1.5–4)	−9.1	0.132	0.138	small
Polyunsaturated Fat (% energy)	4.8 (2.3–6.2)	4.1 (3–5.3)	−14.5	0.141	0.138	small
Monounsaturated Fat (g)	5.8 (3–9.7)	5 (3–9.8)	−14.5	0.184	0.125	small
Monounsaturated Fat (% energy)	12.1 (5.5–14.8)	10.6 (7.7–12.9)	−12.5	0.153	0.134	small
Calcium (mg)	93.5 (51.5–173.8)	67.5 (42.5–100)	−27.8	0.000	0.355	moderate
Iron (mg)	3.1 (1.8–4.4)	2.5 (2–3.8)	−19.4	0.001	0.293	small
Vitamin D (mcg)	0.5 (0.2–0.9)	0 (0–0.2)	−100.0	0.000	0.756	large
Sodium (mg)	505 (244.5–900.8)	600 (300–800)	18.8	0.238	0.111	small
Potassium (mg)	791.5 (425–1348.5)	400 (200–887.5)	−49.5	0.000	0.800	large
Folate (mcg)	57 (28.1–92)	35 (20–57.5)	−38.6	0.000	0.463	moderate
Vitamin C (mg)	3.5 (1–24)	5 (0–10)	44.9	0.000	0.507	large

IQR, interquartile range. ^1^ Effect size ≥ 0.1 and <0.3 was considered small, effect size ≥ 0.3 and <0.5 was considered moderate, and effect size ≥ 0.5 was considered large [28].

**Table 2:** Correlation and cross-classification of quartiles analysis comparing nutrient content of 114 meal photographs with the ChatGPT-estimated nutrient content.

	Correlation ^1^	Cross-Classification of Quartiles ^2^			
	Spearman Coefficient	Exact Agreement, %	Exact Agreement + Adjacent, %	Disagreement, %	Extreme Disagreement, %
Energy (kcal)	0.73	48.2	88.6	11.4	0.0
Protein (g)	0.81	57.9	94.7	5.3	0.0
Protein (% energy)	0.78	54.4	93.0	7.0	0.0
Carbohydrate (g)	0.59	38.6	83.3	16.7	0.0
Carbohydrate (% energy)	0.52	38.6	76.3	20.2	3.5
Fat (g)	0.75	53.5	94.7	3.5	1.8
Fat (% energy)	0.79	53.5	94.7	4.4	0.9
Dietary Fibre (g)	0.64	42.1	82.5	17.5	0.0
Sugar (g)	0.75	55.3	88.6	9.6	1.8
Sugar (% energy)	0.84	64.0	93.9	6.1	0.0
Saturated Fat (g)	0.77	52.6	89.5	9.6	0.9
Saturated Fat (% energy)	0.72	57.9	84.2	15.8	0.0
Polyunsaturated Fat (g)	0.38	33.3	74.6	21.9	3.5
Polyunsaturated Fat (% energy)	0.29	30.7	70.2	21.1	8.8
Monounsaturated Fat (g)	0.61	51.8	79.8	16.7	3.5
Monounsaturated Fat (% energy)	0.54	47.4	83.3	13.2	3.5
Calcium (mg)	0.39	34.2	76.3	19.3	4.4
Iron (mg)	0.53	39.5	76.3	18.4	5.3
Vitamin D (mcg)	0.31	N/A	N/A	N/A	N/A
Sodium (mg)	0.78	53.5	88.6	10.5	0.9
Potassium (mg)	0.83	52.6	96.5	3.5	0.0
Folate (mcg)	0.29	36.8	72.8	18.4	8.8
Vitamin C (mg)	0.69	52.6	86.8	12.3	0.9

^1^ Correlations were statistically significant (*p* < 0.01) for all nutrients. ^2^ Cross-classification of quartiles was not possible for vitamin D due to the number of meals containing no vitamin D; this is therefore marked with N/A.

## Data Availability

The original data presented in the study are openly available in figshare at https://doi.org/10.6084/m9.figshare.28271003.v1.

## Supplementary Materials

The following supporting information can be downloaded at: https://www.mdpi.com/article/10.3390/nu17040607/s1, Figure S1: Examples of the meal photographs uploaded to ChatGPT.

## References

[1] Gandy J. (2019). Welch, Ailsa Dietary Assessment. Manual of Dietetic Practice.

[2] Elmadfa I., Meyer A.L. (2010). Importance of Food Composition Data to Nutrition and Public Health. Eur. J. Clin. Nutr..

[3] Eldridge A.L., Piernas C., Illner A.-K., Gibney M.J., Gurinović M.A., de Vries J.H.M., Cade J.E. (2018). Evaluation of New Technology-Based Tools for Dietary Intake Assessment-An ILSI Europe Dietary Intake and Exposure Task Force Evaluation. Nutrients.

[4] Esquivel M.K., Lozano C.P. (2024). An Overview of Traditional and Novel Tools to Assess Diet. Am. J. Lifestyle Med..

[5] Boushey C.J., Spoden M., Zhu F.M., Delp E.J., Kerr D.A. (2017). New Mobile Methods for Dietary Assessment: Review of Image-Assisted and Image-Based Dietary Assessment Methods. Proc. Nutr. Soc..

[6] Dalakleidi K.V., Papadelli M., Kapolos I., Papadimitriou K. (2022). Applying Image-Based Food-Recognition Systems on Dietary Assessment: A Systematic Review. Adv. Nutr..

[7] Tahir G.A., Loo C.K. (2021). A Comprehensive Survey of Image-Based Food Recognition and Volume Estimation Methods for Dietary Assessment. Healthcare.

[8] Pollard C.M., Pulker C.E., Meng X., Kerr D.A., Scott J.A. (2015). Who Uses the Internet as a Source of Nutrition and Dietary Information? An Australian Population Perspective. J. Med. Internet Res..

[9] Fassier P., Chhim A.-S., Andreeva V.A., Hercberg S., Latino-Martel P., Pouchieu C., Touvier M. (2016). Seeking Health- and Nutrition-Related Information on the Internet in a Large Population of French Adults: Results of the NutriNet-Santé Study. Br. J. Nutr..

[10] Murakami K., Shinozaki N., Okuhara T., McCaffrey T.A., Livingstone M.B.E. (2024). Prevalence and Correlates of Dietary and Nutrition Information Seeking Through Various Web-Based and Offline Media Sources Among Japanese Adults: Web-Based Cross-Sectional Study. JMIR Public Health Surveill..

[11] Achiam J., Adler S., Agarwal S., Ahmad L., Akkaya I., Aleman F.L., Almeida D., Altenschmidt J., Altman S., OpenAI (2024). GPT-4 Technical Report. arXiv.

[12] Carolan K., Fennelly L., Smeaton A.F. (2024). A Review of Multi-Modal Large Language and Vision Models. arXiv.

[13] Qarajeh A., Tangpanithandee S., Thongprayoon C., Suppadungsuk S., Krisanapan P., Aiumtrakul N., Garcia Valencia O.A., Miao J., Qureshi F., Cheungpasitporn W. (2023). AI-Powered Renal Diet Support: Performance of ChatGPT, Bard AI, and Bing Chat. Clin. Pract..

[14] Haman M., Školník M., Lošťák M. (2024). AI Dietician: Unveiling the Accuracy of ChatGPT’s Nutritional Estimations. Nutrition.

[15] Lo F.P.-W., Qiu J., Wang Z., Chen J., Xiao B., Yuan W., Giannarou S., Frost G., Lo B. (2024). Dietary Assessment with Multimodal ChatGPT: A Systematic Analysis. IEEE J. Biomed. Health Inform..

[16] Irish Universities Nutrition Alliance (IUNA) National Adult Nutrition Survey Summary Report. https://www.iuna.net/surveyreports.

[17] O’Hara C., O’Sullivan A., Gibney E.R. (2022). A Clustering Approach to Meal-Based Analysis of Dietary Intakes Applied to Population and Individual Data. J. Nutr..

[18] O’Hara C., Gibney E.R. (2024). Dietary Intake Assessment Using a Novel, Generic Meal–Based Recall and a 24-Hour Recall: Comparison Study. J. Med. Internet Res..

[19] Lyons O.C., Flynn M.A.T., Corish C.A., Gibney E.R., Kerr M.A., McKenna M.J., McNulty H., McSorley E.M., Nugent A.P., O’Brien C. (2022). Nutrition Policy: Developing Scientific Recommendations for Food-Based Dietary Guidelines for Older Adults Living Independently in Ireland. Proc. Nutr. Soc..

[20] Kearney J.M., O’Sullivan E.J., Rajendram R., Preedy V.R., Patel V.B. (2017). Maternal Nutrition in Ireland: Issues of Public Health Concern. Diet, Nutrition, and Fetal Programming.

[21] Chotwanvirat P., Prachansuwan A., Sridonpai P., Kriengsinyos W. (2024). Automated AI-Based Thai Food Dietary Assessment System: Development and Validation. Curr. Dev. Nutr..

[22] Vasiloglou M.F., Mougiakakou S., Aubry E., Bokelmann A., Fricker R., Gomes F., Guntermann C., Meyer A., Studerus D., Stanga Z. (2018). A Comparative Study on Carbohydrate Estimation: GoCARB vs. Dietitians. Nutrients.

[23] Lu Y., Stathopoulou T., Vasiloglou M.F., Pinault L.F., Kiley C., Spanakis E.K., Mougiakakou S. (2020). goFOODTM: An Artificial Intelligence System for Dietary Assessment. Sensors.

[24] Harris P.A., Taylor R., Minor B.L., Elliott V., Fernandez M., O’Neal L., McLeod L., Delacqua G., Delacqua F., Kirby J. (2019). The REDCap Consortium: Building an International Community of Software Platform Partners. J. Biomed. Inform..

[25] Harris P.A., Taylor R., Thielke R., Payne J., Gonzalez N., Conde J.G. (2009). Research Electronic Data Capture (REDCap)—A Metadata-Driven Methodology and Workflow Process for Providing Translational Research Informatics Support. J. Biomed. Inform..

[26] Timon C.M., Evans K., Kehoe L., Blain R.J., Flynn A., Gibney E.R., Walton J. (2017). Comparison of a Web-Based 24-h Dietary Recall Tool (Foodbook24) to an Interviewer-Led 24-h Dietary Recall. Nutrients.

[27] Lombard M.J., Steyn N.P., Charlton K.E., Senekal M. (2015). Application and Interpretation of Multiple Statistical Tests to Evaluate Validity of Dietary Intake Assessment Methods. Nutr. J..

[28] Cohen J. (1992). A Power Primer. Psychol. Bull..

[29] Koo T.K., Li M.Y. (2016). A Guideline of Selecting and Reporting Intraclass Correlation Coefficients for Reliability Research. J. Chiropr. Med..

[30] Sun H., Zhang K., Lan W., Gu Q., Jiang G., Yang X., Qin W., Han D. (2023). An AI Dietitian for Type 2 Diabetes Mellitus Management Based on Large Language and Image Recognition Models: Preclinical Concept Validation Study. J. Med. Internet Res..

[31] Pouladzadeh P., Shirmohammadi S. (2017). Mobile Multi-Food Recognition Using Deep Learning. ACM Trans. Multimed. Comput. Commun. Appl..

[32] Pambudi A., Rofiq M.A., Anwar Sadat M., Arief Soeleman M., Anggi P.R. Knn Algorithm for Foodstuff Classification Using Hsv Color Space and Feature Extraction. Proceedings of the 2022 International Seminar on Application for Technology of Information and Communication (iSemantic).

[33] Suddul G., Seguin J.F.L. (2023). A Comparative Study of Deep Learning Methods for Food Classification with Images. Food Humanit..

[34] Salesse F., Eldridge A.L., Mak T.N., Gibney E.R. (2024). A Global Analysis of Portion Size Recommendations in Food-Based Dietary Guidelines. Front. Nutr..

[35] Ma P., Lau C.P., Yu N., Li A., Liu P., Wang Q., Sheng J. (2021). Image-Based Nutrient Estimation for Chinese Dishes Using Deep Learning. Food Res. Int..

[36] Shonkoff E., Cara K.C., Pei X., Chung M., Kamath S., Panetta K., Hennessy E. (2023). AI-Based Digital Image Dietary Assessment Methods Compared to Humans and Ground Truth: A Systematic Review. Ann. Med..

[37] Lewis P., Perez E., Piktus A., Petroni F., Karpukhin V., Goyal N., Küttler H., Lewis M., Yih W., Rocktäschel T. (2020). Retrieval-Augmented Generation for Knowledge-Intensive NLP Tasks. Proceedings of the 34th International Conference on Neural Information Processing Systems.

[38] Alkhalaf M., Yu P., Yin M., Deng C. (2024). Applying Generative AI with Retrieval Augmented Generation to Summarize and Extract Key Clinical Information from Electronic Health Records. J. Biomed. Informatics.

[39] Kato S., Waki K., Nakamura S., Osada S., Kobayashi H., Fujita H., Kadowaki T., Ohe K. (2016). Validating the Use of Photos to Measure Dietary Intake: The Method Used by DialBetics, a Smartphone-Based Self-Management System for Diabetes Patients. Diabetol. Int..

[40] De Angelis L., Baglivo F., Arzilli G., Privitera G.P., Ferragina P., Tozzi A.E., Rizzo C. (2023). ChatGPT and the Rise of Large Language Models: The New AI-Driven Infodemic Threat in Public Health. Front. Public Health.

[41] Shah S.B., Thapa S., Acharya A., Rauniyar K., Poudel S., Jain S., Masood A., Naseem U. (2024). Navigating the Web of Disinformation and Misinformation: Large Language Models as Double-Edged Swords. IEEE Access.

