# Supplementary material for: An Evaluation of ChatGPT for Nutrient Content Estimation from Meal Photographs
Source: Nutrients. 2025 Feb 7;17(4):607. doi: 10.3390/nu17040607 (PMC11858203; doi:10.3390/nu17040607)
Supplement: Supplementary file 1 [file nutrients-17-00607-s001.zip › nutrients-3464946-supplementary.pdf]

An Evaluation of ChatGPT for Nutrient Content Estimation from Meal Photographs

Cathal O'Hara [1,*], Gráinne Kent [1], Angela C. Flynn [1], Eileen R. Gibney [2,3] and Claire M. Timon [1]

[1] School of Population Health, Royal College of Surgeons in Ireland (RCSI), D02 YN77 Dublin, Ireland; grainnekent@rcsi.ie (G.K.); angelaflynn@rcsi.ie (A.C.F.); clairetimon@rcsi.ie (C.M.T.)
[2] UCD Institute of Food and Health, University College Dublin, D04 V1W8 Dublin, Ireland; eileen.gibney@ucd.ie
[3] School of Agriculture and Food Science, University College Dublin, D04 V1W8 Dublin, Ireland
* Correspondence: cathalohara@rcsi.ie

Supplementary Material

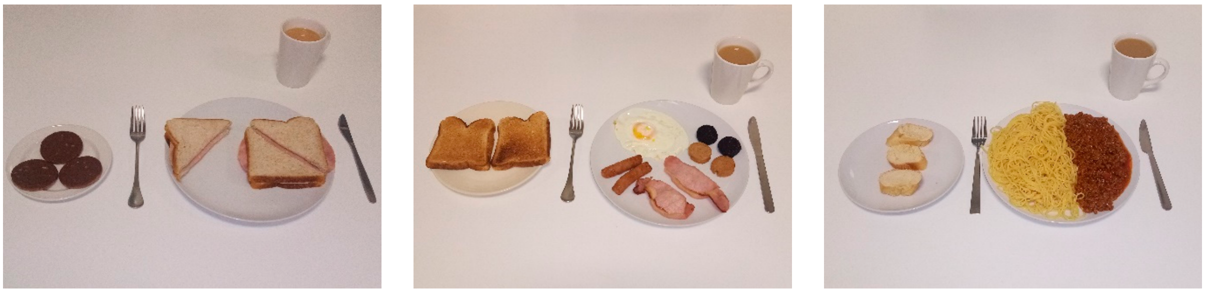

Figure S1: Examples of the meal photographs uploaded to ChatGPT.
